# IAA3-mediated repression of PIF proteins coordinates light and auxin signaling in *Arabidopsis*

**DOI:** 10.1371/journal.pgen.1009384

**Published:** 2021-02-18

**Authors:** Yulin Xi, Yan Yang, Jie Yang, Xing Zhang, Yajie Pan, Hongwei Guo

**Affiliations:** 1 Key Laboratory of Molecular Design for Plant Cell Factory of Guangdong Higher Education Institutes, Department of Biology, Southern University of Science and Technology (SUSTech), Shenzhen, China; 2 The State Key Laboratory of Protein and Plant Gene Research, Peking-Tsinghua Joint Center for Life Sciences, Academy for Advanced Interdisciplinary Studies, School of Life Sciences, Peking University, Beijing, China; 3 Institute of Plant and Food Science, Southern University of Science and Technology (SUSTech), Shenzhen, China; 4 Academy for Advanced Interdisciplinary Studies, Southern University of Science and Technology (SUSTech), Shenzhen, China; National University of Singapore and Temasek Life Sciences Laboratory, SINGAPORE

## Abstract

The exogenous light signal and endogenous auxin are two critical factors that antagonistically regulate hypocotyl growth. However, the regulatory mechanisms integrating light and auxin signaling pathways need further investigation. In this study, we identified a direct link between the light and auxin signaling pathways mediated by the auxin transcriptional repressor IAA3 and light-controlled PIF transcription factors in *Arabidopsis*. The gain-of-function mutation in *IAA3* caused hyposensitivity to light, whereas disruption of *IAA3* led to an elongated hypocotyl under different light intensity conditions, indicating that IAA3 is required in light regulated hypocotyl growth. Genetic studies showed that the function of IAA3 in hypocotyl elongation is dependent on PIFs. Our data further demonstrated that IAA3 interacts with PIFs *in vitro* and *in vivo*, and it attenuates the DNA binding activities of PIFs to the target genes. Moreover, IAA3 negatively regulates the expression of PIFs-dependent genes. Collectively, our study reveals an interplay mechanism of light and auxin on the regulation of hypocotyl growth, coordinated by the IAA3 and PIFs transcriptional regulatory module.

## Author summary

Sessile plants integrate environmental and endogenous signals to optimize their growth and development. Hypocotyl growth is a crucial developmental process tightly affected by light and auxin, but the underlying mechanism is still not well understood. Here, we demonstrate that the IAA3, a suppressor in auxin signaling, negatively regulates the light signaling regulator PIF protein activities. The IAA3 gain-of-function mutant displays reduced responses to light, while disruption of *IAA3* results in elongated hypocotyl under various light intensity conditions. Genetic studies showed that IAA3 functions through PIFs to regulate hypocotyl growth. IAA3 physically interacts with PIFs through its C-terminal region and inhibits PIFs binding to target genes. Furthermore, IAA3 and PIFs coregulated a subset of downstream genes. The IAA3-PIFs interaction represents a novel layer of the regulatory mechanism by which light and auxin signals are integrated to affect hypocotyl growth.

## Introduction

Sessile plants adjust their growth and development in response to the changing environment through a process in which environmental cues and plant intrinsic pathways are tightly integrated [1]. As a vital external signal for plant growth, light provides not only energy but also an informational signal that critically impacts plant development from seed germination to flowering [2–4]. Growing in the soil under natural conditions, *Arabidopsis* seedlings undergo skotomorphogenesis and produce elongated hypocotyls as one of the characteristic features of this process. When the seedlings emerge from the soil, light rapidly stimulates photomorphogenic development, and hypocotyl elongation is consequently inhibited [2,5].

Light signals are sensed by different types of photoreceptors, including phytochromes, cryptochromes, phototropins, and UV Resistance Locus 8 (UVR8) [2], which perceive different wavelengths and intensities of incoming light [6]. These photoreceptors convey the signal to downstream PHYTOCHROME-INTERACTING FACTORs (PIFs, including PIF1, PIF3, PIF4, and PIF5). PIFs are an important subfamily of basic helix-loop-helix (bHLH) transcription factors and act as negative regulators of photomorphogenesis [7]. Among them, PIF3 is the first identified member, and it directly interacts with phyB [8]. Several *PIF3* homologs, including *PIF1*, *PIF4*, and *PIF5*, are also found to regulate photomorphogenesis [9]. The quadruple *PIF* mutant, *pif1 pif3 pif4 pif5* (*pifq*), exhibits a constitutively photomorphogenic phenotype under dark conditions, indicating that these *PIFs* function redundantly to repress photomorphogenesis [10]. PIF proteins accumulate in the dark to maintain skotomorphogenesis. After light irradiation, they are phosphorylated and degraded, leading to photomorphogenetic seedling growth [11]. Several studies have shown how PIF proteins are regulated at the post-transcriptional level. HECATE (HEC) proteins heterodimerize with PIF1 and affect the transcriptional activation activity of PIF1 by preventing DNA binding [12]. Cryptochromes directly interact with PIF4 and PIF5 and modulate their activities in limiting blue light responses [13]. PIF proteins are also involved in diverse internal and external signaling pathways, such as the gibberellin (GA), brassinosteroid (BR), and high temperature pathways [14–18].

Auxin is a core endogenous phytohormone that regulates plant growth and development. In contrast to the light signal, auxin promotes hypocotyl elongation [5,19]. Several auxin biosynthesis deficient or excessive mutants, such as *sav3/taa1* and *yucca*, display defects in hypocotyl elongation [20,21]. It is well established that auxin triggers interaction between the TRANSPORT INHIBITOR RESPONSE 1/AUXIN SIGNALING F-BOX PROTEIN (TIR1/AFB) co-receptor and the AUXIN/INDOLE-3-ACETIC ACID (AUX/IAA) transcriptional repressors, resulting in the degradation of AUX/IAA proteins through the 26S proteasome-mediated pathway. As a result, the inhibition of downstream auxin response factor (ARF) transcription factors are released, and a subset of auxin-responsive genes are activated [22]. There are 29 AUX/IAA proteins in *Arabidopsis*. The typical AUX/IAA proteins contain four conserved domains, I, II, III, and IV [23]. Domain I functions as a repression domain and recruits the TOPLESS (TPL) corepressor for its function. Domain II is required to interact with TIR1/AFB and thus determines the stability of AUX/IAA proteins. Domains III and IV mediate the binding of AUX/IAAs and ARFs [24]. Several mutations in *AUX/IAA* genes have been identified through genetic analysis in *Arabidopsis* [25,26]. Among them, the semidominant *shy2* (*short hypocotyl 2*)/*iaa3* mutations cause shortened hypocotyl and inhibit lateral root development [26–29]. The increasing evidence has demonstrated that IAA3 acts as a node in the regulation of root meristem development by other internal signals, such as cytokinin, brassinosteroids and strigolactones [30–32]. However, the precise role of IAA3 in hypocotyl elongation awaits further investigation.

The interplay between light and auxin contributes to the regulation of a wide range of developmental processes. It was reported that light and auxin co-regulate a number of downstream genes, including the *SMALL AUXIN UPREGULATED* (*SAUR*) genes [33]. Furthermore, several reports have indicated that the light and auxin signaling pathways are integrated by the interaction between the light receptors and several auxin signaling components. For example, one study shows that phyA from oat could interact with several IAA proteins from *Arabidopsis* and pea in an *in vitro* recombinant system [34]. More recently, photoreceptors are shown to interact with IAA proteins to prevent the degradation of IAA proteins mediated by auxin receptors [35,36]. In addition, photoreceptors also target ARF6 and ARF8 to inhibit their DNA-binding activities [37]. Beyond these examples of photoreceptors interacting with IAA or ARF proteins, we wish to explore whether there are additional regulatory modules that directly mediate the crosstalk between the light and auxin signals.

Here, we report that the auxin transcriptional repressor IAA3 regulates hypocotyl elongation by directly modulating PIF protein activities. An *IAA3* gain-of-function mutation led to insensitivity to light, whereas disruption of *IAA3* caused elongated hypocotyl under different light intensity conditions. Genetically, the regulation of IAA3 in hypocotyl elongation is dependent on PIFs. Notably, IAA3 negatively regulates the expression of PIF target genes, by directly interacting with PIFs to interfere with PIFs binding to DNA. Our findings uncover a novel molecular mechanism in which IAA proteins coordinate with PIFs to repress the transcription of target genes, thereby ensuring precise control of hypocotyl growth in response to light and auxin cues.

## Results

### Gain-of-function mutation in *IAA3* causes hyposensitivity to light

**Fig 1:**
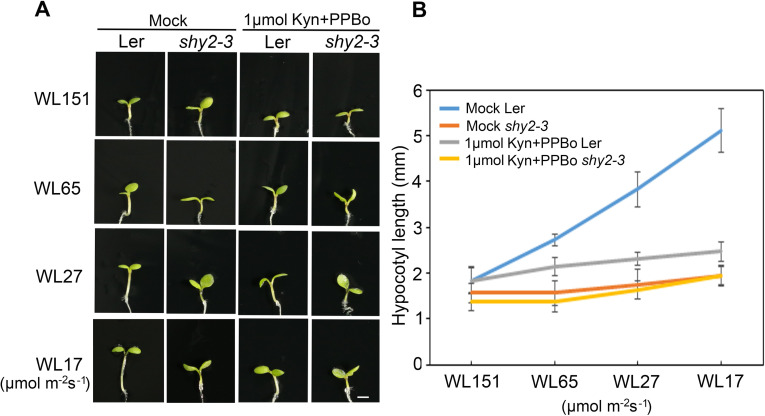
The response of the *shy2-3* mutant to light. (A) Hypocotyl phenotype of 4-day-old seedlings of Ler and shy2-3 mutant under white light (WL) at different fluence rates (151, 65, 27, and 17 μmol·m^−2^·s^−1^) with or without the treatment of auxin synthesis inhibitors (Kyn and PPBo). Bar = 1 mm. (B) Quantification of the hypocotyl length of wild-type L*er* and *shy2-3* under the same conditions described in (A). Error bars represent SD (n ≥ 15 seedlings).

Previous studies showed that a gain-of-function mutation in *IAA3*, *shy2-3*, caused shortened hypocotyl growth. Interestingly, the *shy2-3* mutation could rescue the long hypocotyl phenotype of several light-signaling mutants such as *phyB* [26,29], indicating a possible role of *IAA3* in light signaling to control seedling photomorphogenesis. To assess this putative *IAA3* function, we first examined the light response of the *shy2-3* gain-of-function mutant by treating the seedlings with different light fluence rates. Wild-type L*er* seedlings responded to light normally, with an elongated hypocotyl under low light fluence conditions. In comparison, the hypocotyl of the *shy2-3* mutant displayed a weaker response to light (Fig 1A). Analysis of hypocotyl elongation kinetics revealed that when the light intensity was low, *shy2-3* had reduced hypocotyl elongation compared to wild-type plants (Fig 1B). The hypocotyl response to light in *shy2-3* was similar to that of the *pifq* mutant, which also exhibited insensitivity to light (S1 Fig). Analysis of the *shy2-3* phenotype under dark conditions revealed further similarities to the *pifq* mutant [7,38], as etiolated *shy2-3* seedlings also exhibited a shortened hypocotyl and reduced hook curvature (S2 Fig). As IAA proteins are crucial components in auxin signaling pathway, we further analyzed the effect of auxin on wild type and *shy2-3* mutant. We treated the seedlings with auxin biosynthesis inhibitors Kyn and PPBo to reduce the endogenous auxin level [39,40]. The results showed that the hypocotyl response of wild type plants to light became weakened when auxin was reduced, whereas the *shy2-3* mutant showed no obvious change (Fig 1A and 1B). Together, these results suggest that IAA3 is required for auxin-regulated hypocotyl elongation in light conditions.

### Disruption of *IAA3* leads to elongated hypocotyl

**Fig 2:**
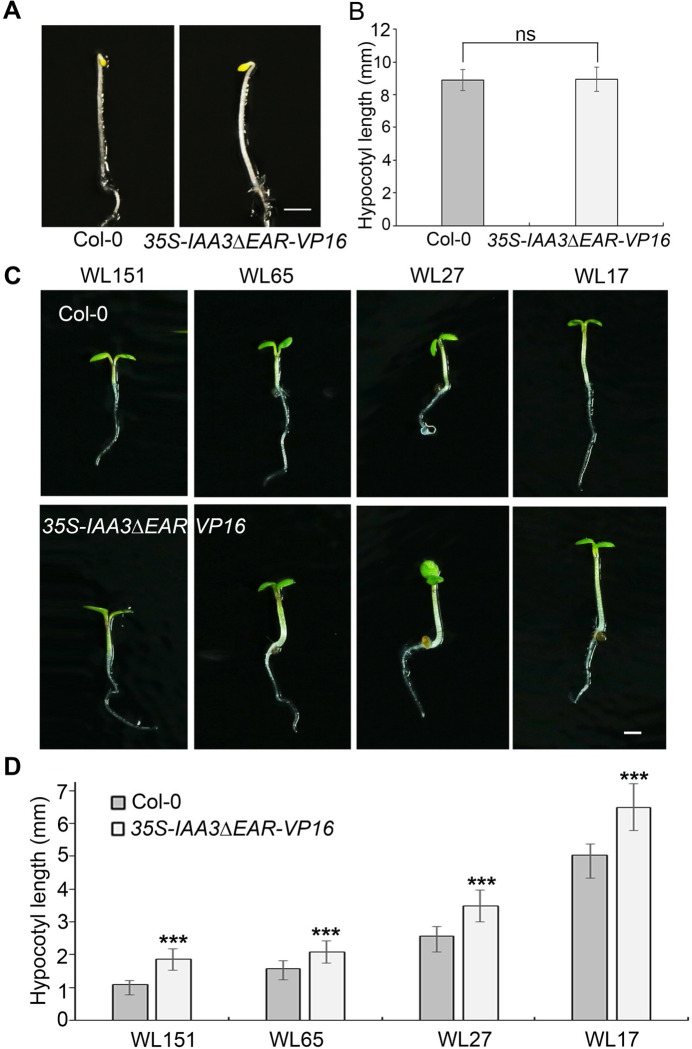
Disruption of *IAA3* causes elongated hypocotyl. (A) Hypocotyl phenotype of 4-day-old etiolated seedlings of the Col-0 and *35S-IAA3ΔEAR-VP16*. Bar = 1mm. (B) Quantification of the hypocotyl length of Col-0, and *35S-IAA3ΔEAR-VP16* under the same conditions described in (A). Error bars represent SD (n ≥ 15 seedlings). Statistical significance was analyzed by the two-tailed Student’s t test (ns represents P > 0.05). (C) Expression of IAA3ΔEAR-VP16 chimeric protein causes hypocotyl elongation at the indicated fluence rates of white light (WL). Four-day-old wild-type Col-0 (top row) and *IAA3ΔEAR-VP16* transgenic plants (bottom row) are shown. Bar = 1mm. (D) Quantification of the hypocotyl length of wild-type Col-0 and *IAA3ΔEAR-VP16* transgenic plants under the same conditions described in (B). Error bars represent SD (n ≥ 15). Significant differences are indicated ***P < 0.001 (Student’s t-test).

*IAA3* belongs to the auxin-induced *AUX/IAA* gene family, whose members often have high functional redundancy. As reported previously, several other IAAs, such as IAA6 and IAA7, may also contribute to hypocotyl elongation [24,25]. To elucidate the function of IAA3 and overcome the possible genetic redundancy, we used a dominant-negative strategy to disrupt the function of the related *AUX/IAA* genes. We generated a *35S-IAA3ΔEAR-VP16* construct by fusing the VP16 transcriptional activation domain to a mutated version of IAA3 lacking the EAR (ERF-associated Amphiphilic Repression) domain to effectively transform the IAA3 transcriptional repressor into an activator. This approach has successfully been used in previous studies to disrupt the redundant functions of multiple repressors [41,42]. We analyzed the hypocotyl elongation of the *35S-IAA3ΔEAR-VP16* transgenic plants under dark and different light intensities. The *35S-IAA3ΔEAR-VP16* transgenic plants displayed similar hypocotyl length with wild type plants in the dark (Fig 2A and 2B). However, compared with wild-type plants, the *35S-IAA3ΔEAR-VP16* transgenic plants showed constitutive longer hypocotyls under different light fluence rates (Fig 2C and 2D), further indicating the important role of IAA3 in light-regulated hypocotyl elongation.

### Genetic interactions between *IAA3* and *PIFs*

The similar hypocotyl phenotypes of *shy2-3* and *pifq* under both light and dark conditions indicated a functional link between IAA3 and PIFs during hypocotyl development. To further reveal the possible functional link between IAA3 and PIFs, we first examined the tissue-specific expression patterns of *IAA3* and *PIFs*. We detected the *IAA3* expression in cotyledon, hypocotyl and root, with the dominant expression in hypocotyl (S3A Fig). Different *PIFs* displayed distinct expression patterns (S3G Fig). In general, both *IAA3* and *PIFs* showed relatively low expression levels in root compared with those in cotyledon and hypocotyl (S3 Fig), suggesting that IAA3 and PIFs express and function primarily in cotyledon and hypocotyl.

**Fig 3:**
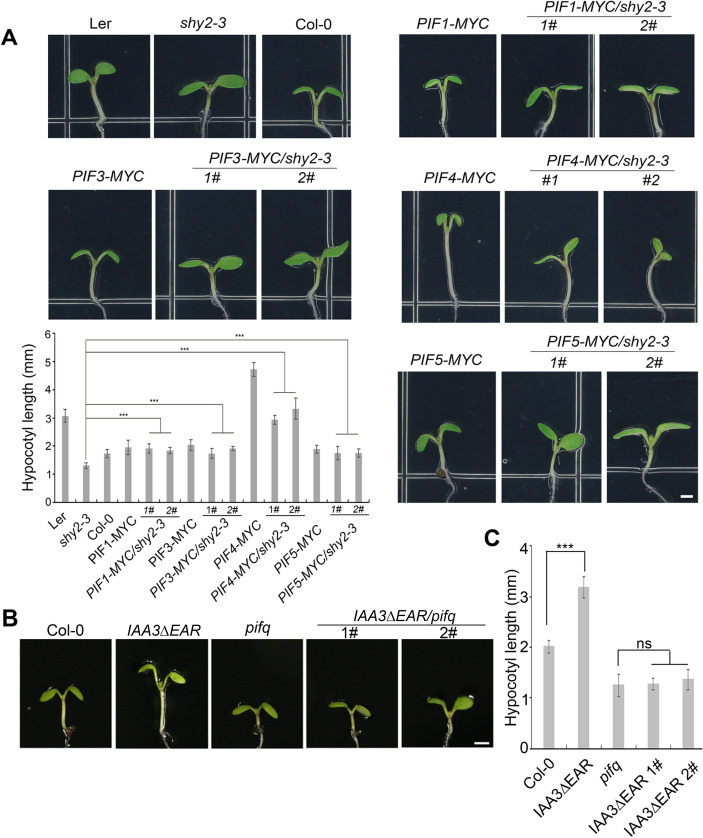
*IAA3* and *PIFs* cooperatively regulate hypocotyl elongation. (A) Overexpression of *PIF* genes rescues the shortened hypocotyl phenotype of *shy2-3*. The images show representative 5-day-old seedlings of L*er*, *shy2-3*, Col-0, *PIF1-MYC*, *PIF1-MYC/shy2-3*, *PIF3-MYC*, *PIF3-MYC/shy2-3*, *PIF4-MYC*, *PIF4-MYC/shy2-3*, *PIF5-MYC*, and *PIF5-MYC/shy2-3*. *PIF-MYC* indicates *35S-PIF-MYC* transgenic plants. The left bottom is the bar graph of the measured hypocotyl lengths of the genotypes indicated. Error bars represent SD (n ≥ 15). Student’s t-tests between mutant/transgenic and mutant seedlings were performed (***P < 0.001). (B) 5-day-old plants of Col-0, *35S-IAA3ΔEAR-VP16* transgenic plant (*IAA3ΔEAR*), *pifq* and the *35S-IAA3ΔEAR-VP16* transgenic line in *pifq* background (*IAA3ΔEAR/pifq*). (C) Bar graph of the measured hypocotyl lengths of the genotypes indicated in (B) Error bars represent SD (n ≥ 15, ***P < 0.001, ns represents P > 0.05).

We then analyzed the genetic interactions between *IAA3* and *PIFs*. We first crossed *shy2-3* with each of the following *35S-PIF-MYC* overexpression lines: *PIF1-MYC*, *PIF3-MYC*, *PIF4-MYC*, and *PIF5-MYC*. The seedlings of all of these *PIF-MYC* lines had elongated hypocotyls compared to wild type when grown under light, but the longest hypocotyls were observed in *PIF4-MYC*. The genetic analysis showed that overexpression of individual *PIFs* significantly rescued the shortened hypocotyl phenotype of the *shy2-3* mutant (Figs 3A and S4). Furthermore, we transformed the *IAA3ΔEAR-VP16* into the *pifq* mutant and analyzed two *IAA3ΔEAR-VP16/pifq* independent lines (S5 Fig). The hypocotyl lengths of *IAA3ΔEAR-VP16/pifq* lines were comparable to those of *pifq* mutants (Fig 3B and 3C), suggesting that the function of IAA3 in hypocotyl regulation is dependent on PIFs.

### IAA3 physically interacts with PIFs

**Fig 4:**
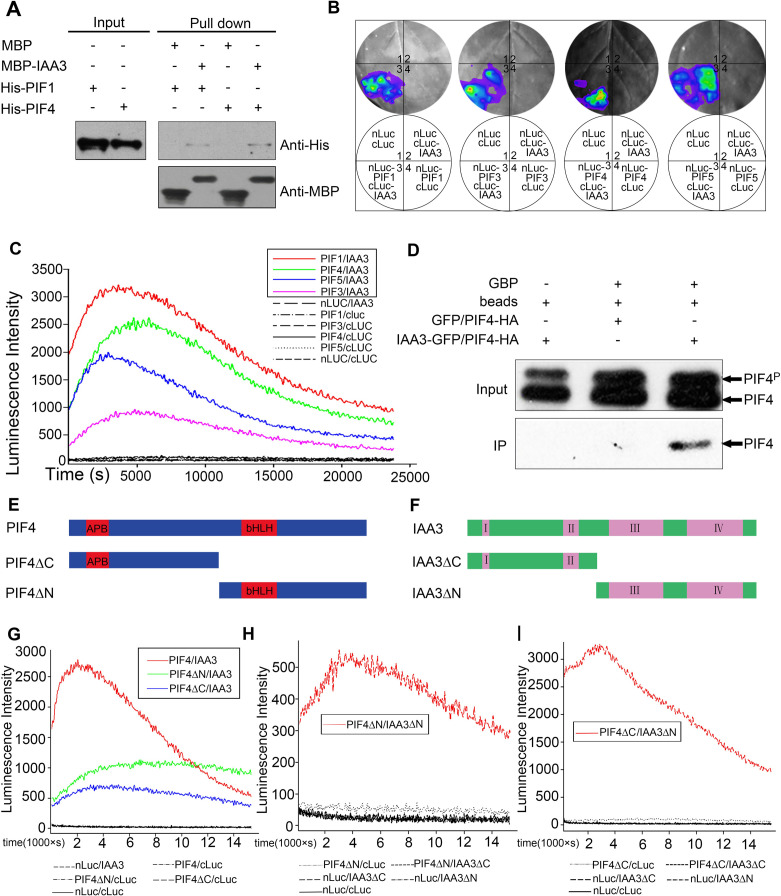
IAA3 interacts with PIFs *in vitro* and *in vivo*. (A) *In vitro* pull-down assays showing that His-PIF1 and His-PIF4 were pulled down by MBP-IAA3 immobilized on amylose resin. (B) and (C) *In vivo* firefly luciferase complementation imaging (LCI) assays in *N*. *benthamiana* leaves (B) and *Arabidopsis* protoplasts (C) confirmed the interactions between IAA3 and PIFs. The full-length *IAA3* sequence was fused to the C-terminal fragment of luciferase (c-LUC), and the full-length sequences of *PIF1*, *PIF3*, *PIF4*, and *PIF5* were individually fused to the N-terminus of luciferase (n-LUC). Empty vectors were used as negative controls. (B) The LUC signals in *N*. *benthamiana* leaves co-infiltrated with different construct pairs are shown in the lower left quadrants of the circles. (C) The listed combinations within the rectangle (PIF1/IAA3, PIF3/IAA3, PIF4/IAA3, and PIF5/IAA3) showed strong interaction; for the other combinations, no interaction was detected. (D) A co-immunoprecipitation (Co-IP) assay indicated that IAA3 could interact with PIF4 when both were transiently expressed in *N*. *benthamiana* leaves. (E) and (F) Domain structures of PIF4 (E), IAA3 (F), and their truncated fragments. The sequences include the full-length PIF4, PIF4ΔC without the bHLH domain, PIF4ΔN without the APB domain (E), full-length IAA3, IAA3ΔC without domains III and IV, and IAA3ΔN without domains I and II (F). (G) Analysis of PIF4 truncated fragments by LCI suggested that both the N- and C-terminals of PIF4 are responsible for the interaction with IAA3. The combinations with strong interactions (PIF4/IAA3, PIF4ΔC/IAA3, and PIF4ΔN/IAA3) are listed in the upper rectangle; negative controls and other combinations that exhibited no interaction are indicated below the graph. (H) and (I) Analysis of IAA3 truncations by LCI indicated that the C-terminal of IAA3 is necessary for the interaction with PIF4. Strong interaction was detected for the combinations listed in the rectangles (PIF4ΔC/IAA3 and PIF4ΔC/IAA3ΔN in (H) and PIF4ΔN/IAA3 and PIF4ΔN/IAA3ΔN in (I); negative controls and other combinations that exhibited no interaction are indicated below the graphs.

The coordination of IAA3 and PIFs in hypocotyl elongation prompted us to investigate whether IAA3 and PIFs physically interact. For *in vitro* pull-down analysis, we co-expressed recombinant His-PIF1 and His-PIF4 with MBP or MBP-IAA3. We detected specific binding of His-tagged PIF1 and PIF4 with MBP-IAA3, with no band detected for the control MBP (Fig 4A), indicating that IAA3 interacted with these PIFs *in vitro*. We then conducted firefly luciferase complementation imaging (LCI) assays to investigate the *in vivo* interactions of IAA3 and PIFs in *N*. *benthamiana* leaves and *Arabidopsis* protoplasts. Luciferase activity signals were detected between IAA3 and PIFs (PIF1, PIF3, PIF4, and PIF5) in both *N*. *benthamiana* leaves and *Arabidopsis* protoplasts (Fig 4B and 4C). Finally, co-immunoprecipitation (Co-IP) experiments using tobacco plants co-expressing IAA3-GFP and PIF4-HA further confirmed that IAA3 interacts with PIF4 *in vivo*. Notably, we found that only the unphosphorylated form of PIF4 protein was immunoprecipitated by IAA3 (Fig 4D). We therefore used LCI analysis to compare the affinity of IAA3 with the unphosphorylated form (designated PIF3 A20) and phosphorylated form of PIF3 (designated PIF3 D19), in which the light-induced phosphorylation sites were mutated to Ala or the phosphomimic mutations were introduced as previously described [43]. Consistent with the findings for PIF4, the LCI analysis indicated preferential binding of IAA3 to the unphosphorylated form of PIF3 (S6 Fig). Collectively, these *in vitro* and *in vivo* studies demonstrated that IAA3 physically interacts with PIFs at the protein level and preferentially binds to unphosphorylated PIFs.

*PIF* genes encode several important domains including an APB domain for phytochrome binding in the N-terminal and a bHLH domain for DNA binding in the C-terminal [44]. We generated two truncated forms of PIF4 to identify the regions of the protein required for interaction with IAA3 (Fig 4E). We found that both the PIF4ΔC without the bHLH domain and PIF4ΔN without the APB domain are responsible for interaction with IAA3 (Fig 4G). We also made truncated forms of IAA3 with only the N-terminal (IAA3ΔC) and C-terminal (IAA3ΔN) regions (Fig 4F). The results showed that only IAA3ΔN (lacking domains I and II) could interact with PIF4ΔN or PIF4ΔC (Fig 4H and 4I), indicating that the IAA3 C-terminal region containing domains III and IV is crucial for the interaction with PIF4.

We also tested the ability of PIF4 to interact with several other members of the AUX/IAA family. The analysis showed that PIF4 also interacted with IAA7, IAA10, IAA15, IAA26, IAA32, and IAA33 (S7 Fig), indicating that multiple AUX/IAA family members may function redundantly to cooperate with PIFs to control plant growth and development.

### IAA3 inhibits PIFs binding to DNA

Based on the finding that IAA3 directly interacts with PIFs, we investigated whether changes in IAA3 protein abundance affected PIF accumulation in plants. Western blot analysis showed that PIF4 protein accumulation was not discernibly altered in the *shy2-3* background (S8A Fig). In addition, there were no significant changes in *PIF* transcript levels after auxin treatment (S8B Fig), indicating that IAA3 may not affect PIF protein accumulation or *PIF* transcript levels.

**Fig 5:**
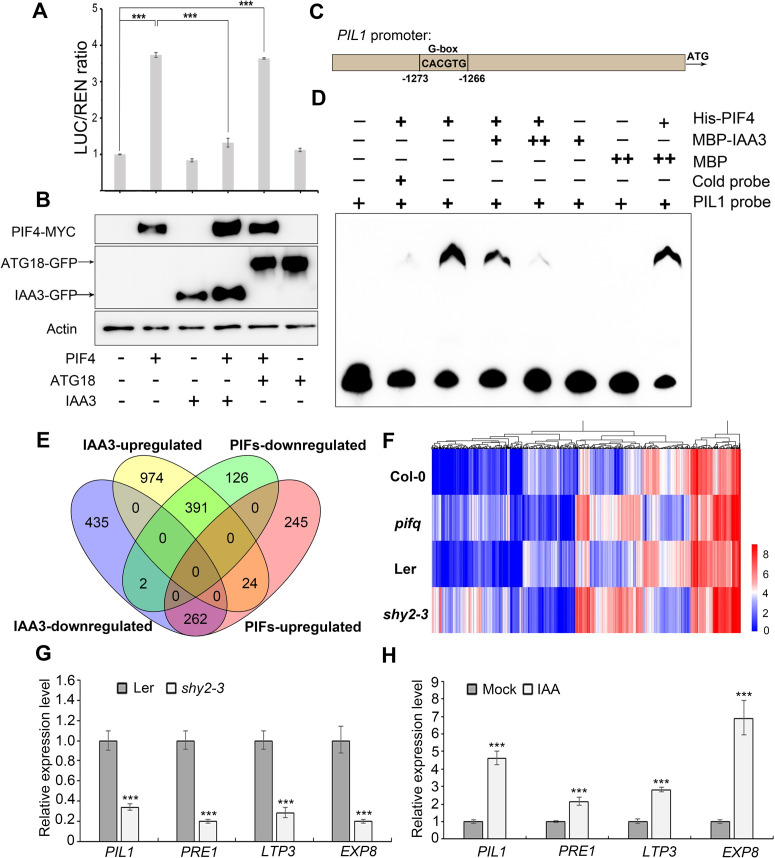
IAA3 impairs PIFs binding to DNA and negatively regulates PIFs’ target genes. (A) The transient dual-LUC reporter assay showing the effects of IAA3 on PIF transcriptional regulation of *PIL1*. The ATG18-GFP protein is as the negative control in the assay. Data are means ± SD (n = 3 experiments). Significant differences are indicated with asterisks (***P < 0.001, two-tailed Student’s t-test). (B) Western blotting for the detection of expressed PIF4-MYC, IAA3-GFP and ATG18-GFP in transformed tobacco leaves from (A) using an anti-MYC or anti-GFP antibody, respectively. (C) A fragment for *PIL1* promoter containing G-box motif (CACGTG) was selected to used as probe in the EMSA experiment. (D) EMSA experiment showing IAA3 inhibition of the DNA-binding activity of PIFs. The cold probe was added as a competitor. MBP was used as a negative control. (E) Venn diagrams showing significant overlap between IAA3-regulated genes and PIFs-dependent genes. Detailed gene information is given in S1 Table. (F) Heat map of 625 DEGs coregulated by IAA3 and PIFs. (G) and (H) qRT-PCR analysis of known PIFs’ direct target genes. The transcription levels of PIFs’ direct target genes in Ler and *shy2-3* mutant (G). Auxin induction of PIFs-regulated genes. Five-day Col-0 seedlings grown in light condition were treated with 5 μM IAA for 24 hours (H). The expression was normalized to the ACTIN2 (*ACT2*) expression level, and was relative to wild-type levels. Data represents mean ±SD from three biological replicates. Significant differences are indicated by ***P < 0.001(two-tailed Student’s t-test).

IAA3 was originally identified as a transcriptional repressor in the auxin signaling pathway [27]. To test whether IAA3 regulates the transcriptional activities of PIFs, we performed a transient expression assay using the reporter construct *ProPIL1*::*LUC*, in which the *LUCIFERASE* (*LUC*) gene was fused with the promoter region of *PIL1*, a direct target gene of PIF [45,46]. LUC activity analysis indicated that PIF4 alone directly activated the expression of *PIL1*, whereas IAA3 alone had no significant effect on its expression (Fig 5A). Remarkably, coexpression of IAA3 and PIF4 significantly repressed the activation executed by PIF4 (Fig 5A). The IAA3 and PIF4 protein levels were monitored by immunoblotting to ensure the comparable expression in each combination (Fig 5B). These results indicate that IAA3 interferes with the PIF transcriptional activity.

Together with our finding in LCI assay that IAA3 interacts with the DNA binding domain of PIF4, we then explored whether IAA3 interacts with PIFs to directly inhibit their DNA-binding ability. The electrophoretic mobility shift assay (EMSA) was conducted with purified recombinant His-PIF4 and MBP-IAA3 proteins. The promoter sequence of a PIF4 target gene, *PIL1*, was used as the binding probe (Fig 5C). As expected, His-PIF4 specifically bound to the promoter of *PIL1* (Fig 5D). Notably, such binding was progressively diminished when increasing amount of IAA3 protein was added (Fig 5D), supporting that IAA3 directly inhibits PIFs binding to their target genes.

### IAA3 negatively regulates the expression of PIFs’ target genes

To further investigate the gene regulatory networks of IAA3 and PIFs, we conducted RNA-seq transcriptome analysis of both *shy2-3* and *pifq* seedlings. We found that 2088 genes and 1050 genes were differentially expressed in the *shy2-3* and *pifq* mutant, respectively (q value<0.05; fold change≥2 and ≤2) (Fig 5E and S1 Table). Of the1050 PIFs-regulated genes, 653 genes (62%) were oppositely affected by *IAA3* (Fig 5E). The Heat map analysis also showed those genes display the similar pattern of FPKM value in *shy2-3* and *pifq* mutants compared with that of in wild type (Fig 5F). Based on the repressive effect of IAA3 to PIFs, we then focused on the 262 genes that were activated by PIFs but repressed by IAA3. Gene ontology (GO) analysis of these genes showed that many auxin-responsive genes, such as *SAURs*, are enriched (S9A and S9B Fig). In addition, we also found several PIFs direct target genes were downregulated in *shy2-3* mutant, such as *PIL1*, *PRE1*, *LTP3* and *EXP8* [45–48]. Quantitative RT-PCR analysis confirmed that the expression levels of these PIFs’ target genes were significantly lower in the *shy2-3* mutant than in wild-type controls (Fig 5G). Consistently, these genes were significantly upregulated in *35S-IAA3ΔEAR-VP16* lines (S10 Fig). Interestingly, we found those PIFs’ target genes could be significantly upregulated by auxin (Fig 5H). Taken together, these results indicate that IAA3 negatively modulates the PIFs-dependent gene expression.

### PIFs are required for auxin-mediated hypocotyl elongation

**Fig 6:**
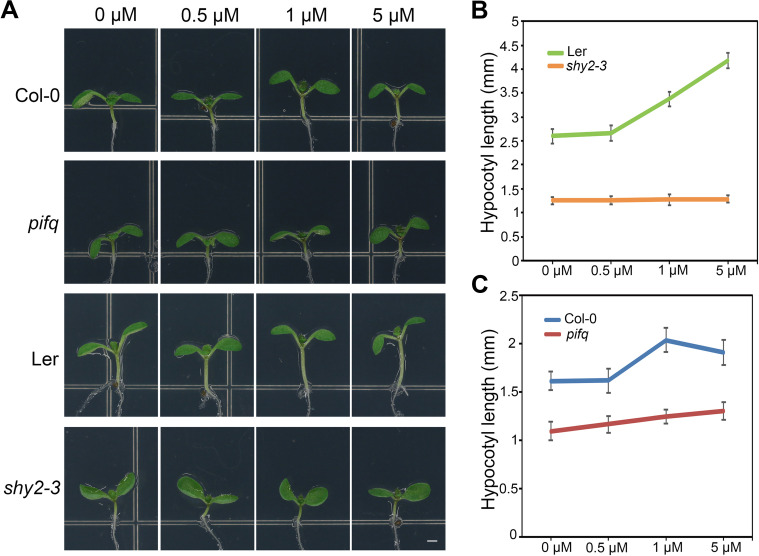
The auxin response in hypocotyl requires PIFs. (A) and (B) The analysis of auxin-regulated hypocotyl elongation indicated that PIFs are required for this growth response. Hypocotyl length of 5-day-old Col, *pifq*, L*er*, and *shy2-3* seedlings grown under long-day conditions and treated with IAA at the indicated concentrations.

The activation of transcriptional auxin signaling pathways is essential for the auxin-mediated hypocotyl elongation response [49]. Based on our findings that PIFs also regulate genes that are responsive to auxin (S9 Fig), we then determined whether PIFs are required for auxin-mediated hypocotyl elongation. Similar to other *AUX/IAA* gain-of-function mutants, the *shy2-3* hypocotyl was insensitive to auxin treatment, which otherwise enhanced the hypocotyl elongation in wild-type plants (Fig 6A and 6B). Remarkably, as in the *shy2-3* mutant, the hypocotyl of the *pifq* mutant also exhibited pronounced resistance to auxin (Fig 6A and 6C). These results support that PIFs are required for auxin-mediated hypocotyl elongation, consistent with the previous report that PIF4 and PIF5 affect hypocotyl sensitivity to auxin [50].

## Discussion

The growth responses of sessile plants to fluctuating environmental conditions must be precise and adaptive. Hypocotyl growth is a developmental event with high plasticity, and it is antagonistically modulated by environmental light and endogenous auxin signals [5]. However, how light and auxin are integrated to regulate hypocotyl growth remains largely unknown. IAAs and PIFs are transcriptional regulators that negatively regulate the auxin and light signaling pathways, respectively [10,23]. In this study, we discovered that IAA3 represses the DNA-binding activity of PIFs through physical interaction, thereby coordinating the light and auxin signals to control hypocotyl elongation.

Several previous reports suggested a close relationship between IAA3 and PIFs. One study indicated that IAA3 is involved in PIF4-mediated high temperature-induced hypocotyl growth [51]. Moreover, IAA3 and PIFs co-regulated a great amount of genes [19]. However, a direct connection between IAA3 and PIFs has not previously been established. In this study, we found that IAA3 directly interacts with multiple PIFs and impairs the binding of PIFs to their target gene promoters (Figs 4 and 5). Notably, we observed preferential binding of IAA3 to unphosphorylated forms of PIFs (Figs 4D and S6). This suggests that even if PIFs are not sufficiently phosphorylated to be degraded under specific conditions, they may nevertheless be subject to repressive regulation by other factors such as IAAs. Under natural conditions, different plant organs may sense and respond to environmental signals such as light in different ways [33]. Thus, the alternative regulation of phosphorylated or unphosphorylated PIFs may be an adaptation allowing specific responses in different organs to the natural environment: compared to the hypocotyl, young leaves may expand to perceive more light, resulting in a greater abundance of the phosphorylated form of PIFs in the leaves and abundant unphosphorylated PIFs in the hypocotyl. In this model, the preferential binding of IAA3 to unphosphorylated PIFs would allow for precise regulation of hypocotyl growth.

It has been reported that IAA proteins could inhibit the DNA-binding activities of ARFs [52,53]. Our finding showed that IAA3 interacts with the DNA binding domain of PIF4 (Fig 4G) and inhibits PIF activity by interfering with their binding to target DNA (Fig 5D). The previous studies also reported that IAA proteins regulate ARF activities by recruiting the TPL/TPR co-repressors [54]. Accordingly, IAA3 may also repress the transcriptional activities of PIFs by recruiting TPL. It is possible that these two mechanisms cooperate together to precisely regulate PIF activities and hypocotyl elongation in response to light and auxin signals.

Although the four *PIFs* function redundantly to regulate the hypocotyl elongation, they may have distinct contributions for hypocotyl regulation. *PIF4* showed a relatively higher expression in hypocotyl than other *PIFs* (S3G Fig), and the overexpression of *PIF4* lines showed the longest hypocotyls compared with that in other *PIFs* overexpression lines (Fig 3A). These results imply that PIF4 might play a dominant role in hypocotyl regulation, thus resulting in the highest degree of *shy2-3* hypocotyl phenotypic rescue by *PIF4* overexpression. Furthermore, IAA3 may display distinct binding ability with the different PIF members, probably a stronger binding ability with PIF1 compared with other PIFs (Fig 4C). As reported previously, PIF1 plays a predominant role in repressing seed germination [55]. It will be interesting to explore whether and how seed germination is regulated by IAA3 and PIF1 transcriptional regulatory module.

ARFs are typical transcription factors that act downstream of IAAs to respond to the auxin signal [56]. Previous reports have indicated that IAA3 associates with ARF7 and ARF19 to regulate root growth [28]. Several ARFs also function redundantly to regulate hypocotyl elongation, and probably through interaction with PIFs or BZR1 [19,57]. Interestingly, the analysis of tissue specific expression of *IAA3* and *PIFs* indicated that *IAA3* and *PIFs* may coexist primarily in cotyledon and hypocotyl (S3 Fig). This also indicates a specificity of signal integration achieved through IAA3 interaction with different types of transcription factors in specific tissues.

**Fig 7:**
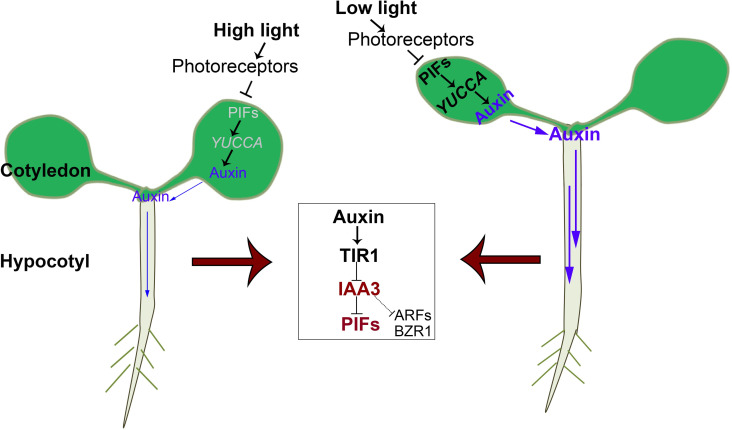
A working model of how different light conditions and auxin regulate seedling growth through the functions of IAAs and PIFs. The different responses of specific seedling tissues to changing light conditions and auxin reflect different patterns of IAA and PIF activity in these tissues. In young leaves, low light is perceived by photoreceptors and then triggers the accumulation of PIFs, leading to increased *YUCCA* expression and auxin abundance. The auxin in the leaves is transported to the hypocotyl, in which IAA3 is degraded through the TIR1-mediated proteasome pathway, releasing the inhibition of several transcription factors including PIFs, ARFs, and BZR1. The target genes of these transcription factors are activated to promote hypocotyl elongation. When the light conditions are sufficiently high, PIF accumulation in the leaves is reduced, leading to decreased *YUCCA* expression, and then reduced auxin levels in the leaves. Thus, the auxin in the hypocotyl which is transported from leaves is also reduced. Consequently, IAA3 represses PIFs, ARFs, and BZR1 in the hypocotyl. The blue arrows in the diagram represent auxin flow and the thickness of the arrows represent auxin level.

Auxin synthesis is considered to occur in the cotyledon, followed by transport from the shoot to the root [5]. Light is a key factor affecting cotyledon auxin levels, as it impacts PIFs accumulation as well as *YUCCA* transcript levels [58]. On the basis of previous reports and the results of the present study, we propose a model that illustrates how light and auxin signals be integrated in specific tissues (Fig 7). Low light signals trigger auxin synthesis in the cotyledon, which is mediated by a transcriptional regulatory module consisting of PIFs and *YUCCA*. Auxin is subsequently transported to promote hypocotyl elongation, which is mediated by the interaction between IAAs and different types of transcription factors, including PIFs, ARFs, and BZR1.

Our data showed that PIF4 could also interact with several non-canonical IAA proteins (S7 Fig), such as IAA32 and IAA33, which lack the conserved domains I and II [24]. Mechanistically, these non-canonical IAAs may function distinctly from canonical IAAs. IAA32 was recently identified as a target of transmembrane kinase 1 (TMK1) in the regulation of apical hook development [59]. As previously reported, PIFs also affect apical hook development [38]. Thus, the interaction between IAA32 and PIF4 observed in the present study is consistent with their involvement in apical hook development, most likely as downstream components in the TMK1-mediated signaling pathway. Alternatively, since different AUX/IAA family members function redundantly during hypocotyl elongation [25,26], these PIF-interacting non-canonical IAAs may also regulate hypocotyl elongation. The functions of these non-canonical IAAs in hypocotyl elongation warrant further study, along with the connection between non-canonical and canonical IAAs.

## Methods

### Plant materials and growth conditions

All *Arabidopsis thaliana* mutants and transgenic lines used in this study except *shy2-3* are in the Col-0 background. *shy2-3* is in the L*er* background [27]. *pif1-1 pif3-3 pif4-2 pif5-3* (*pifq*) [60], *35S-PIF4-MYC* (abbreviated as *PIF4-MYC*), and *shy2-3* were lab stock. *35S-PIF1-MYC* (abbreviated as *PIF1-MYC*), *35S-PIF3-MYC* (abbreviated as *PIF3-MYC*), *35S-PIF3-D19-MYC* (abbreviated as *PIF3 D19*), and *35S-PIF5-MYC* (abbreviated as *PIF5-MYC*) transgenic lines were provided by Peter H. Quail. *PIF1-MYC/shy2-3*, *PIF3-MYC/shy2-3*, *PIF4-MYC/shy2-3*, and *PIF5-MYC/shy2-3* were generated by crossing, followed by kanamycin selection and PCR-based genotyping of the F2 population. To generate *35S-IAA3ΔEAR-VP16*, *IAA3* without the EAR motif was amplified, and the PCR product was cloned into pENTR/D-TOPO to generate pENTR-IAA3*Δ*EAR. The *VP16* coding region was amplified from pENTR-R2-VP16-L3 [41] then fused to IAA3*Δ*EAR using restriction enzymes to generate pENTR-IAA3*Δ*EAR-VP16. The 35S-IAA3ΔEAR-VP16 construct was generated by LR reactions between plasmids pK2GW7 and pENTR-IAA3*Δ*EAR-VP16. This construct was transformed into *Agrobacterium tumefaciens* GV3101/pMP90 by electroporation and then into *Arabidopsis* using the floral dip method [61].

Surface-sterilized seeds were sown on Murashige and Skoog (MS) medium (4.4 g/L MS salt, 1% sucrose, pH 5.7, and 0.8% agar) and imbibed for 3 d at 4°C in the dark. Light-grown seedlings were grown under white fluorescent light (about 130 μmol m^-2^s^-1^) with a 16 h light/8 h dark photoperiod unless otherwise indicated. Dark-grown seedlings were exposed to white light for 4 h then transferred to dark conditions. Hypocotyl lengths were measured from the intersection of the cotyledons to the root using ImageJ software, and more than 15 seedlings were measured for each set of experiments.

### RNA extraction, reverse transcription, and real-time PCR

Total RNA was extracted from seedlings using Trizol reagent (Invitrogen). Total RNA was subjected to reverse transcription at 42°C for 1 h with M-MLV reverse transcriptase (Promega). Real-time PCR was performed on a Light Cycler 480 system (Roche) with SYBR Premix Ex Taq reagent (TaKaRa). Each experiment was performed at least three times using different pools of seedlings under the indicated conditions.

### Firefly luciferase complementation assay

The coding sequences of *PIF1/3/4/5*, *PIF4ΔN*, and *PIF4ΔC* were individually inserted into the multiple cloning site of pCAMBIA1300-nLUC, and the coding sequences of *IAA3*, *IAA3ΔN*, and *IAA3ΔC* were inserted into the multiple cloning site of pCAMBIA1300-cLUC [62]. Different vector combinations were transformed into *Agrobacterium* strain GV3101, which was subsequently infiltrated into tobacco leaves. After infiltration, the tobacco leaves were grown under weak light for 12 h then transferred to light conditions for 48 h. The abaxial sides of leaves were sprayed with 1 mM luciferin, followed by a 10 min incubation in the dark. Luciferase activity was recorded on a Xenogen IVIS Spectrum instrument.

For transformation into *Arabidopsis* protoplasts, plasmids were extracted and purified using Plasmid Maxiprep kits (Vigorous). *Arabidopsis* protoplasts were prepared from 10-day-old green seedlings or 4-week-old adult seedlings following a standard protocol. Ten micrograms of both the nluc-tagged and cluc-tagged constructs were co-transformed into protoplasts and incubated for 12 to 16 h in darkness. After the incubation period, luciferin (Gold Biotechnology) was added, and luciferase activity was recorded on an LB 985 NightSHADE system (Berthold Technologies).

### Transient transcription activation assay

The *ProPIL1*::*LUC* reporter was constructed by amplifying 1.8 kb of the *Arabidopsis PIL1* promoter and inserting the fragment into the pGreen II 0800-LUC vector. The coding sequences of *PIF4*, *IAA3*, and *ATG18* were amplified and inserted into the PQG110 vector to generate effector constructs. The DLR system (Promega) was used for transient expression analysis in leaves of tobacco [63]. The activities of firefly (*Photinus pyralis*) luciferase and *Renilla reniformis* luciferase (LUC and REN, respectively) were sequentially measured from a single sample on a GLO-MAX 20/20 luminometer (Promega). The ratio of LUC/REN was calculated as an indicator of the final transcriptional activity.

### Protein extraction and immunoblotting

Whole seedlings were finely ground in liquid nitrogen and suspended thoroughly in protein extraction buffer (60 mM Tris-HCl, pH 6.8, 25% glycerol, 2% SDS, 14.4 M β-mercaptoethanol, 0.1% bromophenol, and 1 M DTT) by vortexing. Proteins were extracted by boiling for 10 min at 100°C then roughly separated via centrifugation (13,000 rpm for 15 min at 4°C). Supernatants were loaded onto 10% SDS-PAGE gels, and the proteins were transferred to a nitrocellulose filter membrane (Millipore) according to a standard protocol. Anti-MYC (ABclonal) was diluted 5000-fold for incubation with the membranes. Goat anti-mouse HRP-conjugated secondary antibody (Promega) was diluted 7500-fold.

### Co-immunoprecipitation assay

For Co-IP experiments, the 35S-IAA3-GFP and 35S-PIF4-HA constructs were transformed into *Agrobacterium* strain GV3101, which was subsequently infiltrated into tobacco leaves. After infiltration, the tobacco leaves were grown under weak light for 12 h then transferred to light conditions for 48 h. Soluble and nuclear proteins from tobacco leaves were isolated using IP buffer (50 mM Tris-HCl, pH 8.0, 150 mM NaCl, 10% glycerol, 0.5 mM EDTA, 0.1% Triton X-100, 5mM β-mercaptoethanol, and 1× cocktail). The soluble proteins from tobacco leaves co-expressing 35S-GFP and 35S-PIF4-HA or 35S-IAA3-GFP and 35S-PIF4-HA were immunoprecipitated with GFP-binding protein (GBP, a gift from Professor Junyu Xiao of Peking University) that binds Ni-NTA Agarose (Qiagen). The mixtures were maintained at 4°C with gentle shaking for 3 h then washed with cold IP buffer three times. The samples were detected by anti-HA (Qiagen) and anti-GFP antibodies (Abmart).

### Protein expression and purification

The coding sequences of *PIF1* and *PIF4* were cloned into the pCold-TF vector (GE Healthcare) and transformed into Transette (DE3) competent cells (TransGen). Protein expression was induced by 0.1 mM IPTG at 16°C, and proteins were purified with Ni-NTA Agarose (Qiagen) following the manufacturer’s instructions. The coding sequence of *IAA3* was cloned into pMAL-c2X vector (NEB) then transformed into Transette (DE3) competent cells (TransGen). Protein expression was induced by 0.4 mM IPTG at 16°C, and proteins were purified with amylose agarose (NEB) following the manufacturer’s instructions.

### Pull-down assay

The in vitro-purified MBP and MBP-IAA3 were incubated with Amylose Resin (NEB) in pull-down buffer (50 mM Tris-Cl, pH 8.0, 150 mM NaCl, 10% glycerol, 0.5 mM EDTA, 0.1% Triton X-100, 5 mM β-mercaptoethanol, and 1× protease inhibitor cocktail) for 4 h at 4°C. The fusion protein His-PIF1 and His-PIF4 was added and incubated for another 3 h at 4°C. After five washes with pull-down buffer, the precipitated resins were collected by brief centrifugation then treated with protein extraction buffer. Proteins were separated by SDS-PAGE and detected with anti-MBP (NEB) or anti-His (Tiangen).

### EMSA assay

The oligonucleotide probes for *PIL1* were synthesized with the sequence information described previously [46]. The *PIL1* probe was labeled with digoxigenin (DIG) by company. The LightShift Chemiluminescent EMSA Kit (Pierce) was used in the assay. The biotin-labeled probe was incubated with the proteins in 1× binding buffer for 60 min. A 500-fold excess of cold probe was added to the reaction system for competition. The 6% polyacrylamide gel was used for electrophoresis.

### RNA sequencing

The five-day-old seedlings of Ler, *shy2-3*, Col-0 and *pifq* plants grown in light were collected to extract RNA for RNA sequencing using the Illumina HiSeq X Ten system in biotechnology company. The bioinformatic and statistical analysis of the RNA-seq data was performed as described previously [64]. Genes with changes of more than two-fold (Q-value ≤ 0.05) were defined as differentially expressed genes.

## Supporting information

S1 Table Genes regulated by IAA3 and PIFs. (XLSX)

S1 Fig The response of the *pifq* mutant to light. (A) Hypocotyl phenotype of 4-day-old seedlings of the Col-0 and *pifq* mutant under continuous white light (WL) at different fluence rates (151, 65, 27, and 17 μmol·m^−2^·s^−1^). Bar = 1 mm. (B) Quantification of the hypocotyl length of wild-type wild-type Col-0 and *pifq* under the same conditions described in (A). Error bars represent SD (n ≥ 15).
(TIF)

S2 Fig The phenotype of *shy2-3* in dark condition. (A) Hypocotyl and apical hook phenotype of 4-day-old etiolated seedlings of wild type Ler and *shy2-3* mutant. Bar = 1mm. (B) and (C) Quantification of the hypocotyl length and hook curvature of L*er* and *shy2-3* in (A). Error bars represent SD (n ≥ 15). Significant differences are indicated ***P < 0.001 (Student’s t-test).
(TIF)

S3 Fig The tissue specific expression of *IAA3* and *PIFs*. (A) to (G) qRT-PCR analysis of *IAA3* and *PIFs* in cotyledon, hypocotyl and root, respectively. Data represents mean ±SD from three biological replicates.
(TIF)

S4 Fig The phenotype of *PIFs-MYC/shy2-3* in dark condition. (A) Hypocotyl phenotype of 4-day-old etiolated seedlings of the indicated genotypes. Bar = 1mm. (B) and (C) Quantification of the hypocotyl length of in Col-0, *shy2-*3, *PIFs-MYC* and *PIFs-MYC/shy2-3* in (A). Error bars represent SD (n ≥ 15). Significant differences are indicated ***P < 0.001 (Student’s t-test).
(TIF)

S5 Fig The expression of *IAA3ΔEAR* in *IAA3ΔEAR/pifq*. (A) and (B) The mRNA level of *IAA3ΔEAR* in Col-0, *IAA3ΔEAR*, *pifq* and the *IAA3ΔEAR/pifq*. Data represents mean ±SD from three biological replicates. Significant differences are indicated **P < 0.01, ***P < 0.001 (two-tailed Student’s t-test).
(TIF)

S6 Fig IAA3 prefers to interact with the unphosphorylated form of PIF3. Interactions between IAA3 and unphosphorylated PIF3 (PIF3 A20) or phosphorylated PIF3 (PIF3 D19) by LCI analysis in *Arabidopsis* protoplasts.
(TIF)

S7 Fig PIF4 interacts with multiple IAA proteins. Interaction between PIF4 and other AUX/IAA protein members, including IAA7, IAA10, IAA15, IAA26, IAA32, and IAA33, by LCI analysis in *Arabidopsis* protoplasts. PIF4 showed a strong interaction with these IAA proteins but showed no interaction with the empty vectors.
(TIF)

S8 Fig Analyses of PIF protein accumulation and *PIF* transcript levels. (A) PIF4 protein levels in 5-day-old *PIF4-MYC* and *PIF4-MYC/shy2-3* seedlings. (B) Transcript levels of *PIF* genes in 5-day-old Col-0 seedlings treated with the indicated concentrations of auxin for 3 hours. Data represents mean ±SD from three biological replicates. Statistical significance was analyzed by the two-tailed Student’s t test (ns represents P > 0.05).
(TIF)

S9 Fig The GO analysis of IAA3-repressed and PIFs-activated genes. (A) The GO analysis of IAA3 repressed and PIFs activated genes. (B) The FPKM value of *SAURs* in RNA-seq data.
(TIF)

S10 Fig The expression of IAA3-regulated genes in *35S-IAA3ΔEAR-VP16*. qRT-PCR analysis of *PIL1*, *PRE1*, *LTP3* and *EXP8* in wild type Col-0 and 35S-*IAA3ΔEAR-VP16* transgenic plants. Data represents mean ±SD from three biological replicates. Significant differences are indicated by ***P < 0.001 (two-tailed Student’s t-test).
(TIF)
