# Supplementary figures and images for: IAA3-mediated repression of PIF proteins coordinates light and auxin signaling in Arabidopsis
Source: PLoS Genet. 2021 Feb 18;17(2):e1009384. doi: 10.1371/journal.pgen.1009384 (PMC7924758; doi:10.1371/journal.pgen.1009384)

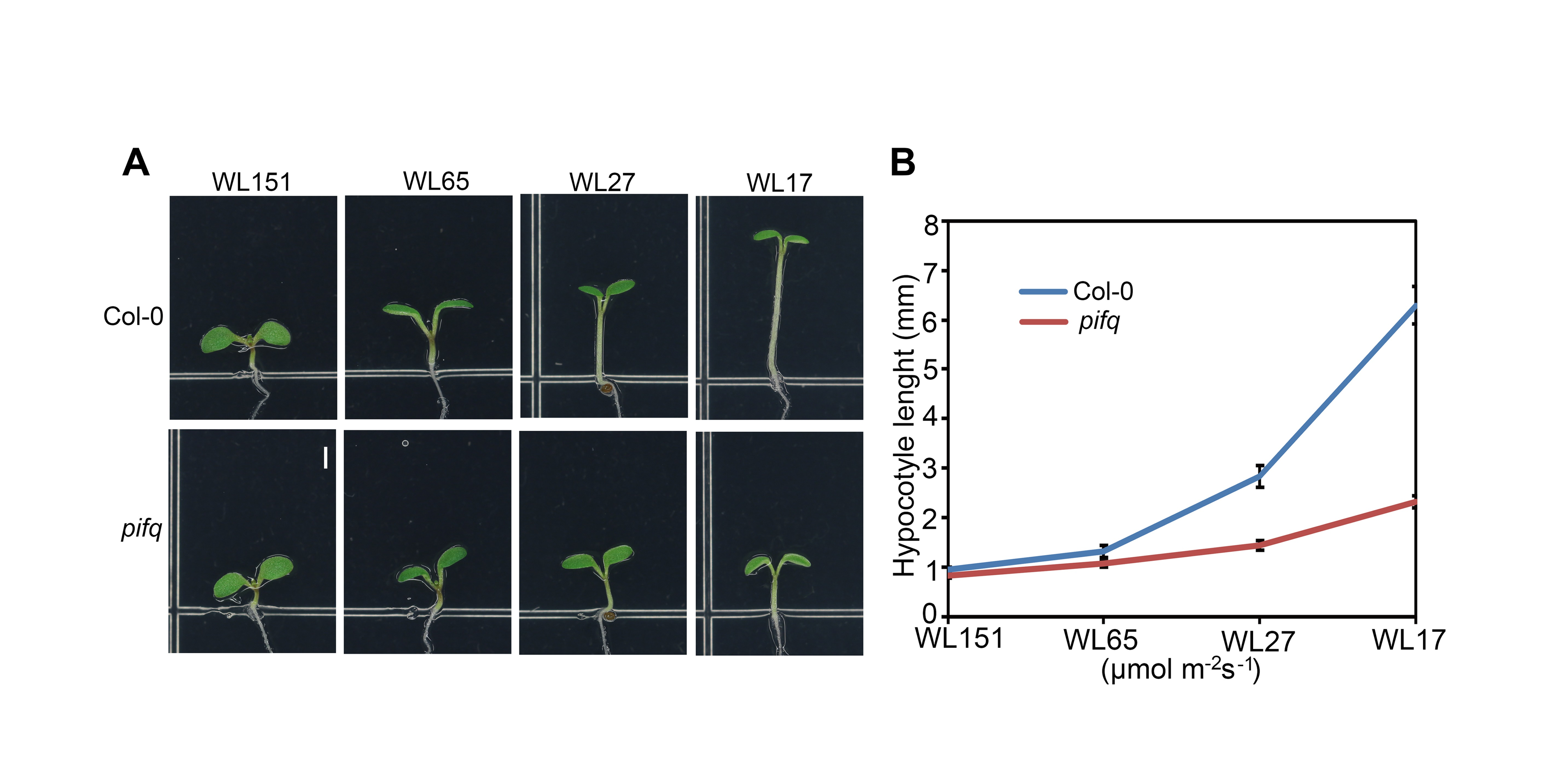

Supplement: S1 Fig — (A) Hypocotyl phenotype of 4-day-old seedlings of the Col-0 and pifq mutant under continuous white light (WL) at different fluence rates (151, 65, 27, and 17 μmol·m−2·s−1). Bar = 1 mm. (B) Quantification of the hypocotyl length of wild-type wild-type Col-0 and pifq under the same conditions described in (A). Error bars represent SD (n ≥ 15). (TIF) [file pgen.1009384.s002.tif]

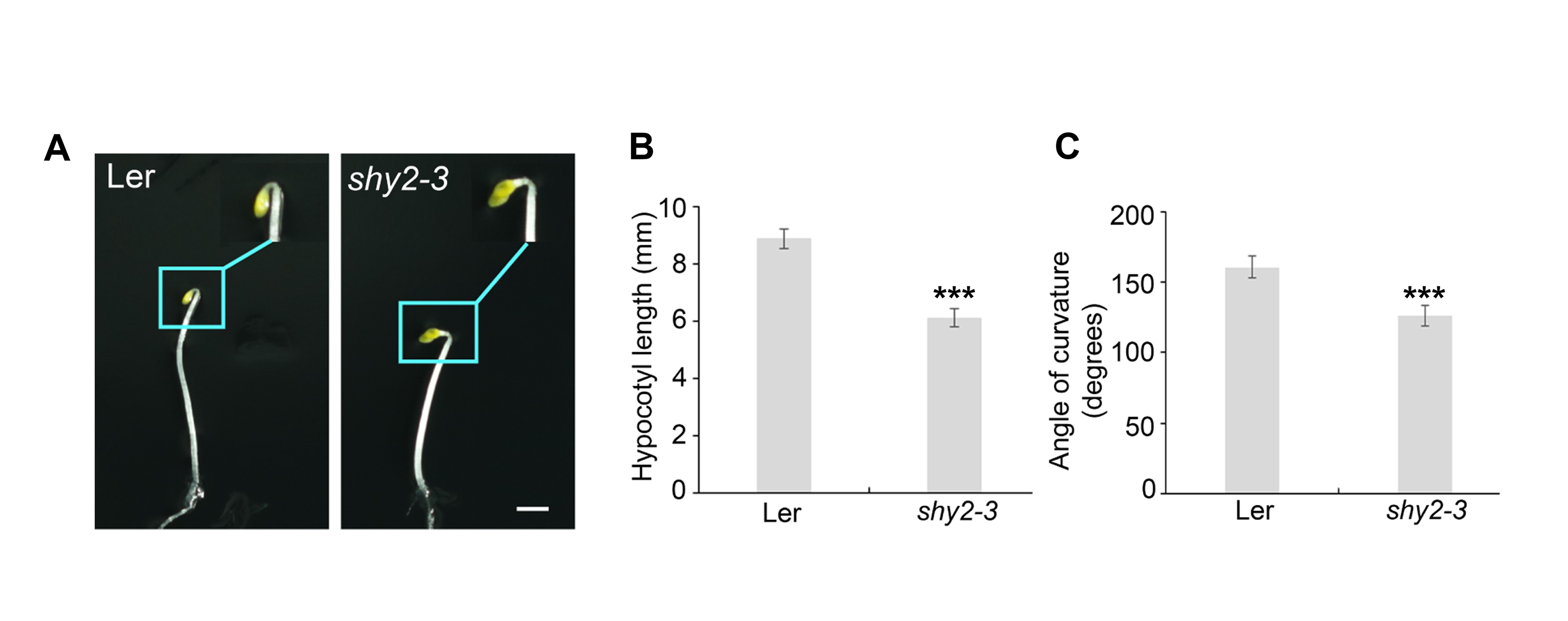

Supplement: S2 Fig — (A) Hypocotyl and apical hook phenotype of 4-day-old etiolated seedlings of wild type Ler and shy2-3 mutant. Bar = 1mm. (B) and (C) Quantification of the hypocotyl length and hook curvature of Ler and shy2-3 in (A). Error bars represent SD (n ≥ 15). Significant differences are indicated ***P < 0.001 (Student’s t-test). (TIF) [file pgen.1009384.s003.tif]

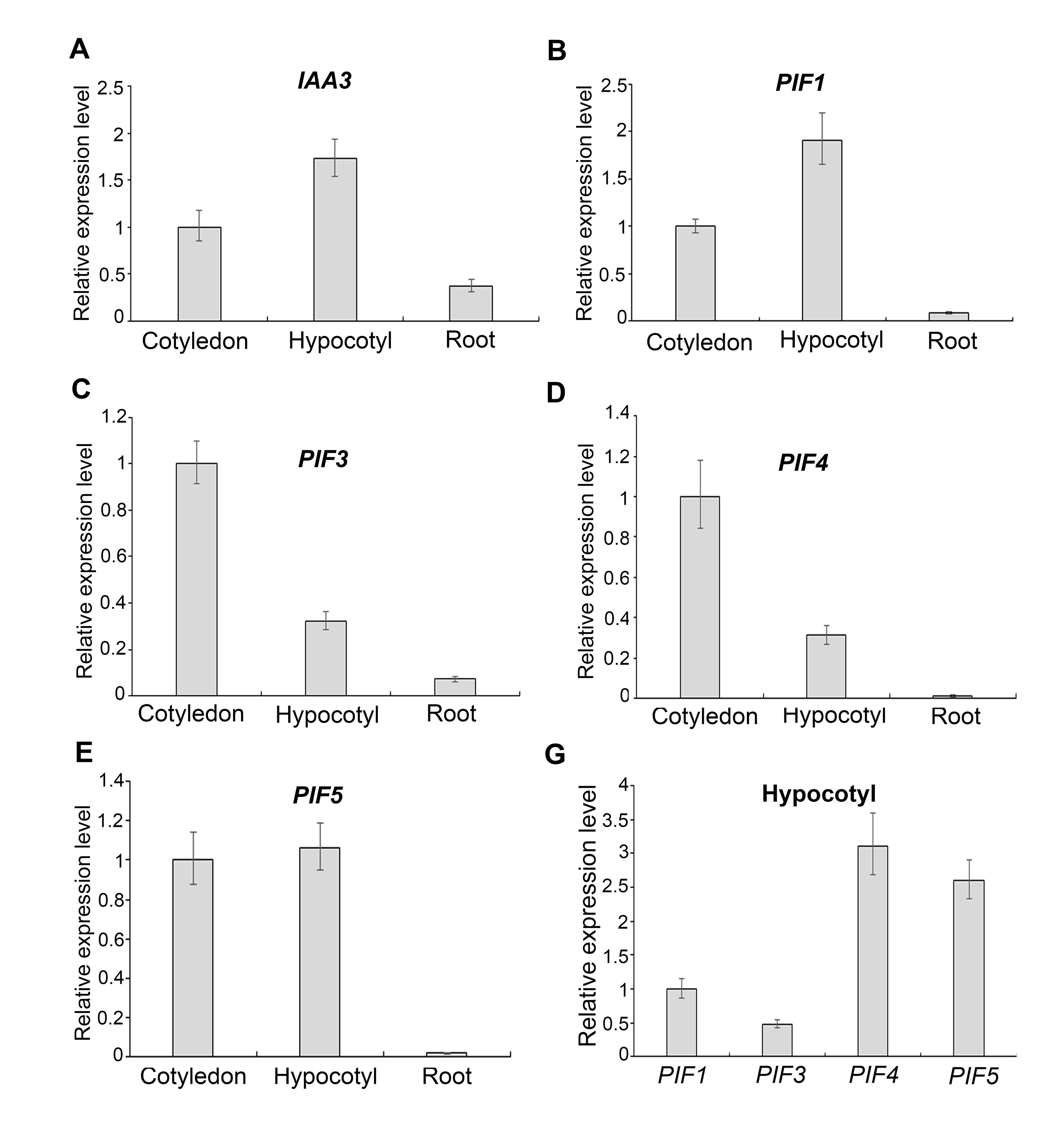

Supplement: S3 Fig — (A) to (G) qRT-PCR analysis of IAA3 and PIFs in cotyledon, hypocotyl and root, respectively. Data represents mean ±SD from three biological replicates. (TIF) [file pgen.1009384.s004.tif]

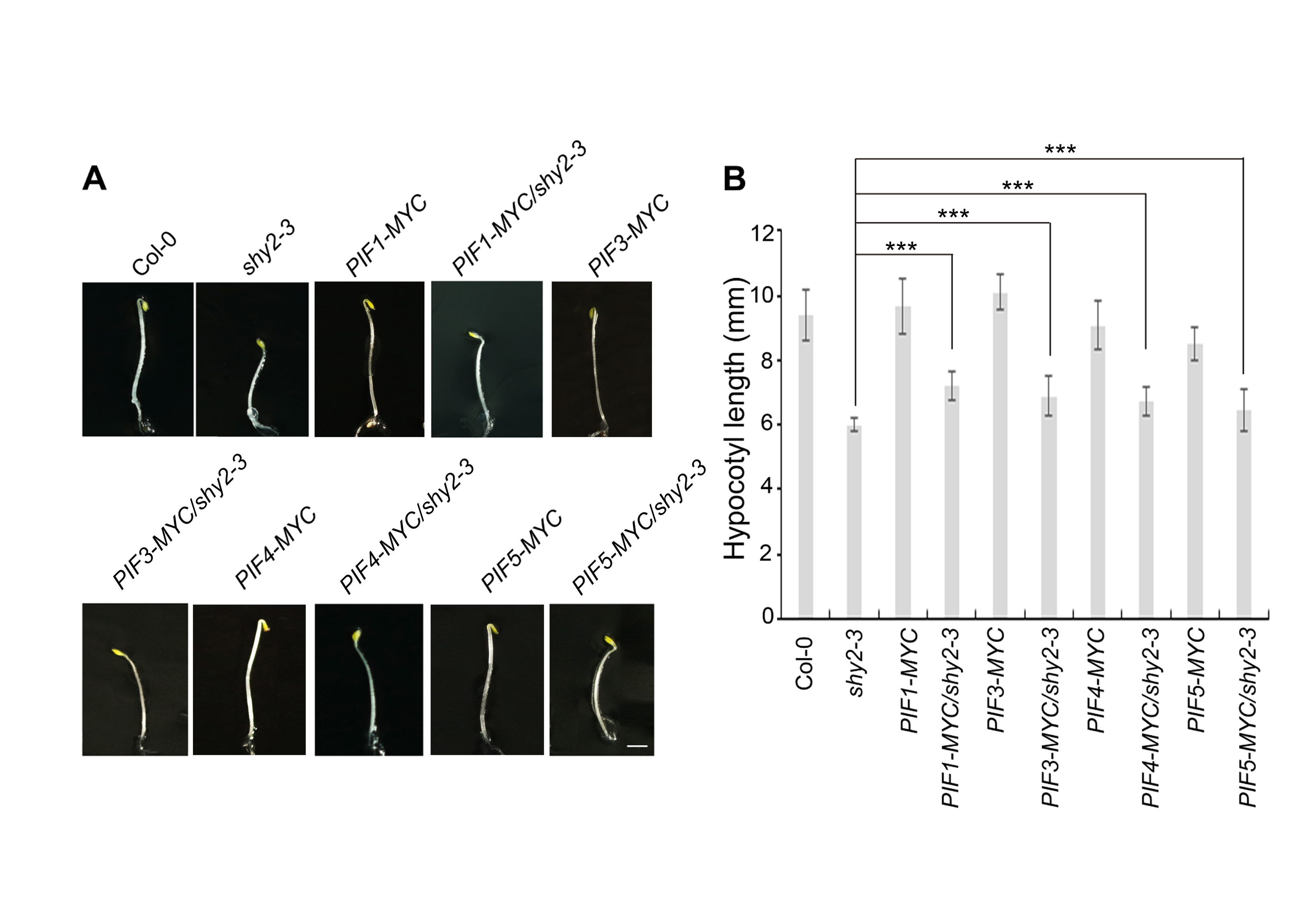

Supplement: S4 Fig — (A) Hypocotyl phenotype of 4-day-old etiolated seedlings of the indicated genotypes. Bar = 1mm. (B) and (C) Quantification of the hypocotyl length of in Col-0, shy2-3, PIFs-MYC and PIFs-MYC/shy2-3 in (A). Error bars represent SD (n ≥ 15). Significant differences are indicated ***P < 0.001 (Student’s t-test). (TIF) [file pgen.1009384.s005.tif]

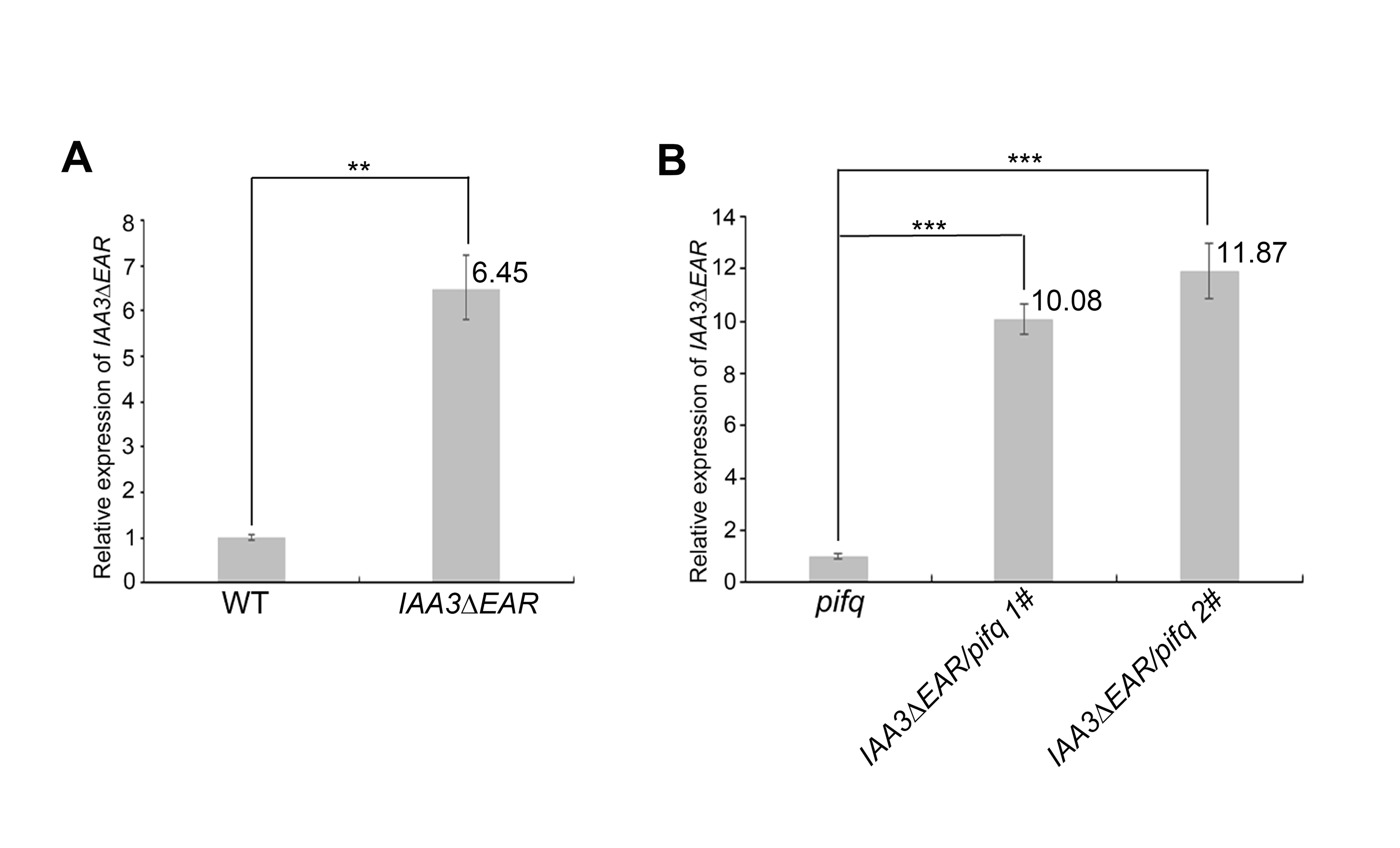

Supplement: S5 Fig — (A) and (B) The mRNA level of IAA3ΔEAR in Col-0, IAA3ΔEAR, pifq and the IAA3ΔEAR/pifq. Data represents mean ±SD from three biological replicates. Significant differences are indicated **P < 0.01, ***P < 0.001 (two-tailed Student’s t-test). (TIF) [file pgen.1009384.s006.tif]

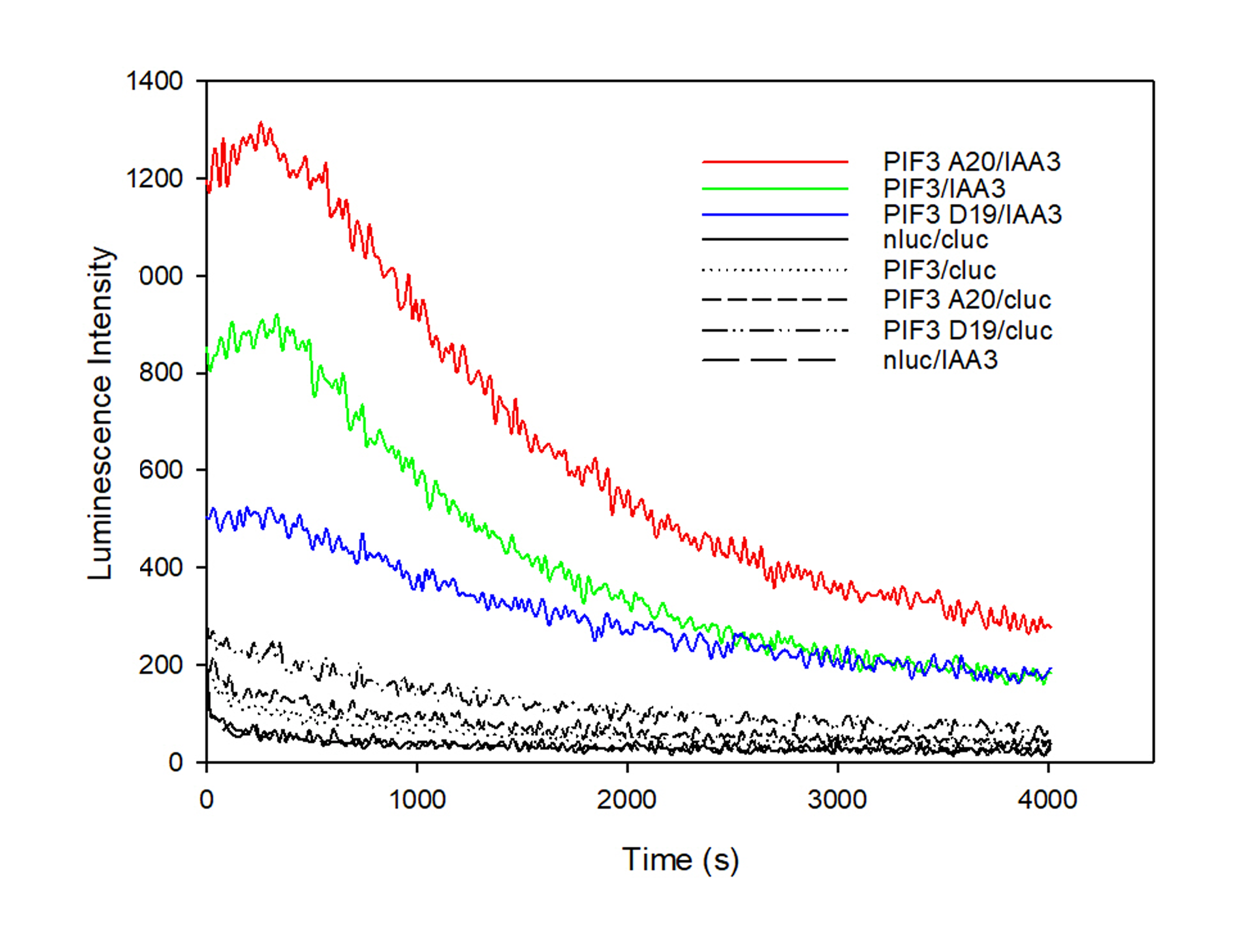

Supplement: S6 Fig — Interactions between IAA3 and unphosphorylated PIF3 (PIF3 A20) or phosphorylated PIF3 (PIF3 D19) by LCI analysis in Arabidopsis protoplasts. (TIF) [file pgen.1009384.s007.tif]

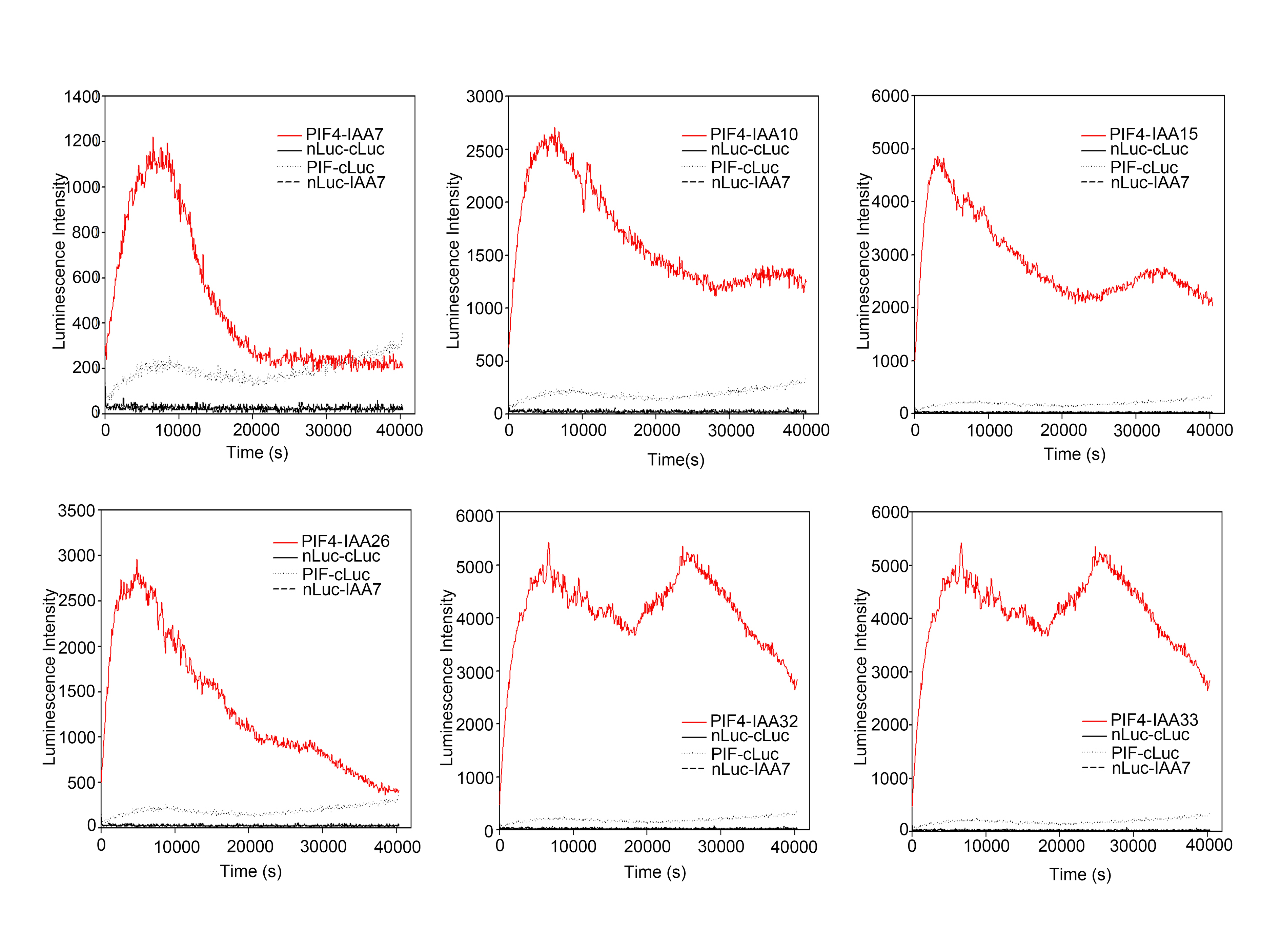

Supplement: S7 Fig — Interaction between PIF4 and other AUX/IAA protein members, including IAA7, IAA10, IAA15, IAA26, IAA32, and IAA33, by LCI analysis in Arabidopsis protoplasts. PIF4 showed a strong interaction with these IAA proteins but showed no interaction with the empty vectors. (TIF) [file pgen.1009384.s008.tif]

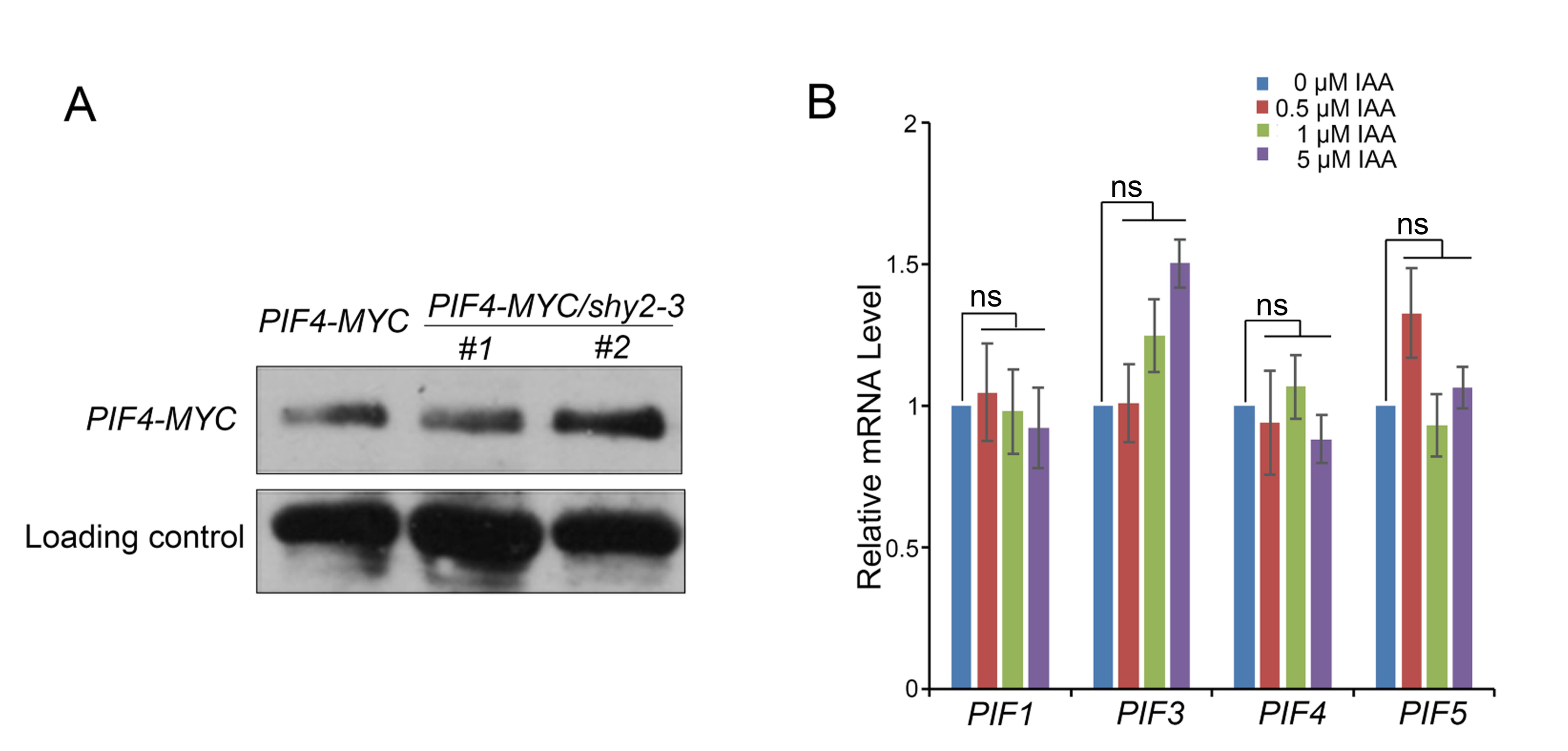

Supplement: S8 Fig — (A) PIF4 protein levels in 5-day-old PIF4-MYC and PIF4-MYC/shy2-3 seedlings. (B) Transcript levels of PIF genes in 5-day-old Col-0 seedlings treated with the indicated concentrations of auxin for 3 hours. Data represents mean ±SD from three biological replicates. Statistical significance was analyzed by the two-tailed Student’s t test (ns represents P > 0.05). (TIF) [file pgen.1009384.s009.tif]

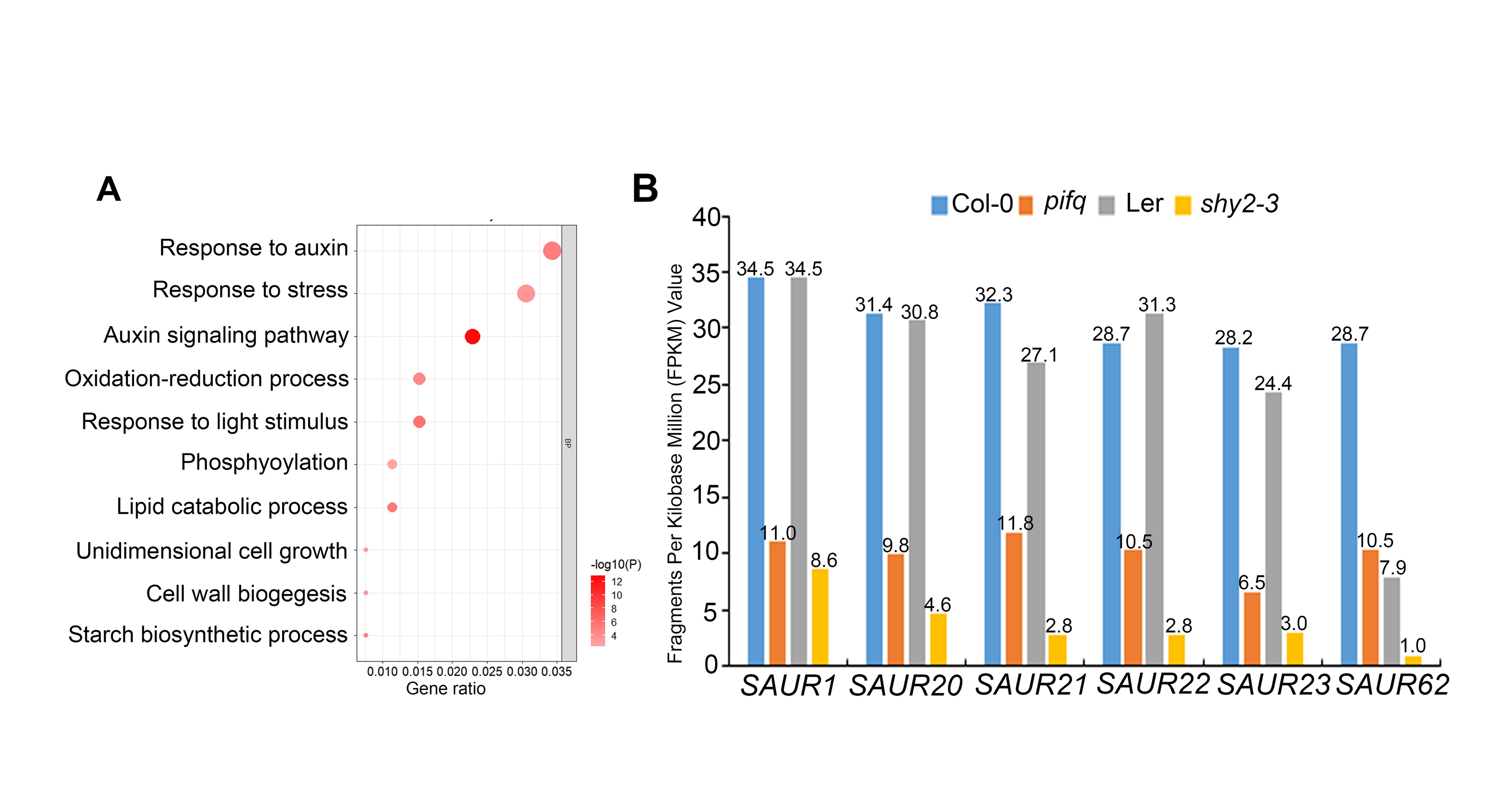

Supplement: S9 Fig — (A) The GO analysis of IAA3 repressed and PIFs activated genes. (B) The FPKM value of SAURs in RNA-seq data. (TIF) [file pgen.1009384.s010.tif]

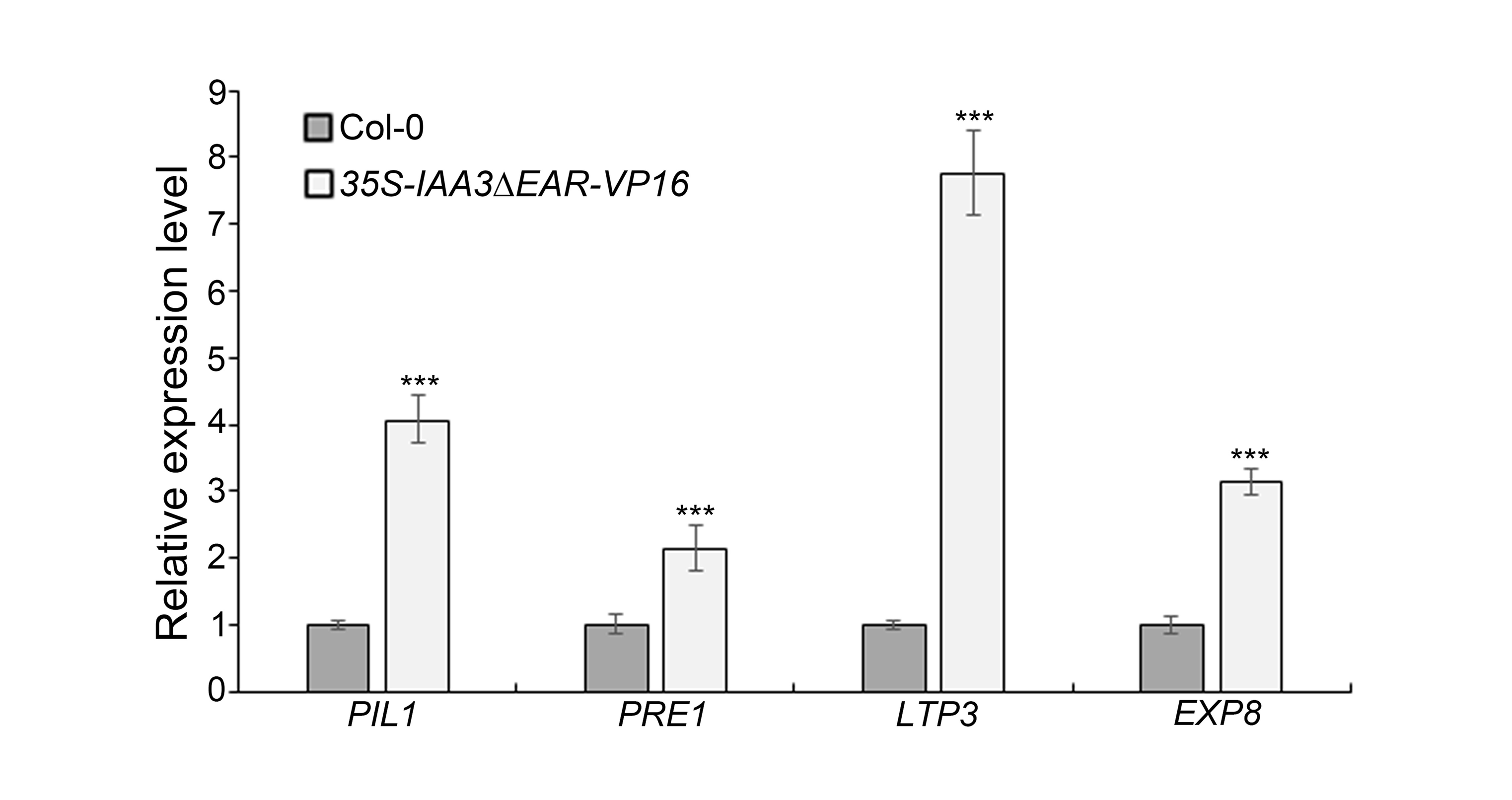

Supplement: S10 Fig — qRT-PCR analysis of PIL1, PRE1, LTP3 and EXP8 in wild type Col-0 and 35S-IAA3ΔEAR-VP16 transgenic plants. Data represents mean ±SD from three biological replicates. Significant differences are indicated by ***P < 0.001 (two-tailed Student’s t-test). (TIF) [file pgen.1009384.s011.tif]
